# Assembly and Annotation of a Draft Genome of the Medicinal Plant *Polygonum cuspidatum*

**DOI:** 10.3389/fpls.2019.01274

**Published:** 2019-10-18

**Authors:** Yonghong Zhang, Lanlan Zheng, Yan Zheng, Chao Zhou, Ping Huang, Xiao Xiao, Yongheng Zhao, Xincai Hao, Zhubing Hu, Qinhua Chen, Hongliang Li, Xuanbin Wang, Kenji Fukushima, Guodong Wang, Chen Li

**Affiliations:** ^1^Laboratory of Medicinal Plant, Institute of Basic Medical Sciences, School of Basic Medicine, Biomedical Research Institute, Hubei Key Laboratory of Wudang Local Chinese Medicine Research, Hubei University of Medicine, Shiyan, China; ^2^Key Laboratory of Three Gorges Regional Plant Genetics and Germplasm Enhancement (CTGU)/Biotechnology Research Center, China Three Gorges University, Yichang, China; ^3^Center for Multi-Omics Research Key Laboratory of Plant Stress Biology, State Key Laboratory of Cotton Biology, School of Life Sciences, Henan University, Kaifeng, China; ^4^Affiliated Dongfeng Hospital, Hubei University of Medicine, Shiyan, China; ^5^Laboratory of Chinese Herbal Pharmacology, Oncology Center, Renmin Hospital, Biomedical Research Institute, Hubei University of Medicine, Shiyan, China; ^6^Institute for Molecular Plant Physiology and Biophysics, University of Würzburg, Würzburg, Germany; ^7^Key Laboratory of Medicinal Resources and Natural Pharmaceutical Chemistry, Ministry of Education, National Engineering Laboratory for Resource Development of Endangered Crude Drugs in Northwest China, College of Life Sciences, Shaanxi Normal University, Xi’an, China

**Keywords:** *Polygonum cuspidatum*, genome assembly, resveratrol biosynthesis, whole-genome duplication, medicinal plant, stress tolerance

## Abstract

*Polygonum cuspidatum* (Japanese knotweed, also known as Huzhang in Chinese), a plant that produces bioactive components such as stilbenes and quinones, has long been recognized as important in traditional Chinese herbal medicine. To better understand the biological features of this plant and to gain genetic insight into the biosynthesis of its natural products, we assembled a draft genome of *P. cuspidatum* using Illumina sequencing technology. The draft genome is ca. 2.56 Gb long, with 71.54% of the genome annotated as transposable elements. Integrated gene prediction suggested that the *P. cuspidatum* genome encodes 55,075 functional genes, including 6,776 gene families that are conserved in the five eudicot species examined and 2,386 that are unique to *P. cuspidatum*. Among the functional genes identified, 4,753 are predicted to encode transcription factors. We traced the gene duplication history of *P. cuspidatum* and determined that it has undergone two whole-genome duplication events about 65 and 6.6 million years ago. Roots are considered the primary medicinal tissue, and transcriptome analysis identified 2,173 genes that were expressed at higher levels in roots compared to aboveground tissues. Detailed phylogenetic analysis demonstrated expansion of the gene family encoding stilbene synthase and chalcone synthase enzymes in the phenylpropanoid metabolic pathway, which is associated with the biosynthesis of resveratrol, a pharmacologically important stilbene. Analysis of the draft genome identified 7 abscisic acid and water deficit stress-induced protein-coding genes and 14 cysteine-rich transmembrane module genes predicted to be involved in stress responses. The draft *de novo* genome assembly produced in this study represents a valuable resource for the molecular characterization of medicinal compounds in *P. cuspidatum*, the improvement of this important medicinal plant, and the exploration of its abiotic stress resistance.

## Introduction

*Polygonum cuspidatum* Sieb. et Zucc., commonly known as Huzhang in Chinese and Japanese knotweed in English, is a medicinal plant that is widely distributed in eastern Asia whose roots have served for centuries as an important traditional Chinese medicine for dispelling wind-evil, damp elimination, analgesic therapy, relieving coughs, and reducing sputum (Peng et al., 2013; Hong et al., 2016). *P. cuspidatum* belongs to the Polygonaceae family of eudicots, which includes many other key medicinal plants, such as *Rheum palmatum* (Chinese rhubarb), *Fagopyrum cymosum* (tall buckwheat), and *Polygonum multiflorum*, as well as the pseudocereal crop *Fagopyrum tataricum* (Tartary buckwheat). In contrast to its medicinal uses, *P. cuspidatum* is regarded as an invasive plant in Europe and North America due to its aggressive growth, allelopathic effects, and extremely strong abiotic stress tolerance (Murrell et al., 2011; Rouifed et al., 2012; Dommanget et al., 2016). This species shows strong adaptability and tolerance to a wide range of stress conditions, such as dense shade, high temperatures, cold, drought, waterlogging, burning, heavy metals, various soil types, and extreme pH, salt, and high sulfur dioxide conditions (Clements and Ditommaso, 2012; Michalet et al., 2017). However, few molecular genetic studies have been conducted on this pharmaceutically and economically important herbaceous plant.

Modern pharmacological studies have indicated that extracts from *P. cuspidatum* have anti-inflammatory, antioxidant, and hepatoprotective properties and could be to treat cancer and other diseases (Zhang et al., 2013; Olaru et al., 2015; Sohn et al., 2015; Abu-Amero et al., 2016; Liu and Huang, 2016; Geng et al., 2018). Extensive photochemistry investigations have led to the isolation and identification of more than 67 compounds from the roots and leaves of *P. cuspidatum*, providing chemical evidence for its pharmacological effect (Vastano et al., 2000; Yi et al., 2007; Shan et al., 2008). The major compounds in *P. cuspidatum* are stilbenes and quinones (Vastano et al., 2000; Shan et al., 2008). The most abundant stilbenes in *P. cuspidatum* are polydatin and resveratrol, which have been identified in several other plants, including grapevine (*Vitis vinifera*) (Langcake and Pryce, 1976), peanuts (*Arachis hypogaea*) (Sanders et al., 2000), blueberries (*Vaccinium* spp.) (Lyons et al., 2003), cocoa (*Theobroma cacao*) (Counet et al., 2006), and sorghum (*Sorghum bicolor*) (Burdette et al., 2010; Vanamala et al., 2017). Resveratrol functions in plant disease resistance by activating constitutive and inducible defense responses (Chong et al., 2009). Resveratrol, especially in the form of *trans*-resveratrol, has many pharmacological uses in treating inflammation, HIV, and cardiovascular-related diseases (Hao et al., 2012; Ouyang et al., 2014; Hong et al., 2016). Although some recent clinical studies have cast doubt on its pharmacological activities, resveratrol has been widely utilized in the nutraceutical and cosmetics industries for decades (Donnez et al., 2009; Lu et al., 2016). The largest portion of resveratrol in the global market is currently produced from the root extracts of field-grown *P. cuspidatum* (Mei et al., 2015).

The anthraquinone emodin and its derivative, physcion, are major quinones in *P. cuspidatum* and have potential antimicrobe and anticancer applications (Jayasuriya et al., 1992; Teich et al., 2004; Su et al., 2005; Olsen et al., 2007; Yang et al., 2007; Shan et al., 2008; Narender et al., 2013; Chen et al., 2015; Xiong et al., 2015; Han et al., 2016; Hong et al., 2016; Li et al., 2016; Liu et al., 2016; Pan et al., 2016; Gao et al., 2017). Physcion significantly inhibits cancer cell proliferation and tumor growth in mice by specifically decreasing the activity of 6-phosphogluconate dehydrogenase, thus affecting the oxidative pentose phosphate pathway (Lin et al., 2015).

Resveratrol is produced by a well-characterized biosynthetic pathway that includes four major enzymes: phenylalanine ammonia lyase (PAL), cinnamic acid 4-hydroxylase (C4H), 4-coumarate CoA ligase (4CL), and stilbene synthase (STS) (Ferrer et al., 1999; Hao et al., 2012; Jiang et al., 2018; Zhao et al., 2018). PAL, C4H, and 4CL are members of the common phenylpropanoid pathway in plants, which synthesizes phenolic compounds. By contrast, STS is a type III polyketide synthase (PKS), which catalyzes the condensation of resveratrol in the final step of the pathway (Hao et al., 2012). Plant type III PKSs have been widely studied, including chalcone synthase (CHS), which plays an important role in plant metabolite biosynthesis (Yu et al., 2012). STS shares high amino acid sequence identity (>70%) with CHS; these enzymes comprise the STS/CHS superfamily (Melchior and Kindl, 1990; Tropf et al., 1995; Ferrer et al., 1999). STS and CHS catalyze the same iterative condensation to yield tetraketide intermediates in the same manner (Austin and Noel, 2003; Funa et al., 2006). Although STS is thought to have arisen from CHS several times during the course of evolution (Tropf et al., 1994), no genome-wide investigation of CHS/STS family genes in *P. cuspidatum* has been conducted thus far. Although CHS and STS share the same substrate in flowering plants, CHS is responsible for the biosynthesis of chalcones, which serve as starting molecules for flavonoid compounds (Schröder et al., 1988; Wang et al., 2017a). While CHS is widely present in many plants, STS is only found in plants that produce resveratrol (Kiselev, 2011).

Several genes that are likely involved in resveratrol biosynthesis have been identified (Ferrer et al., 1999; Hao et al., 2012; Jiang et al., 2018; Zhao et al., 2018), but little is known about the genetic basis of the anthraquinone biosynthetic pathway. Our current understanding on anthraquinone biosynthesis were obtained from the family Rubiaceae and especially in the genera *Rubia* and related species, such as *Rubia cordifolia* (Wijnsma and Verpoorte, 1986; Han et al., 2001; Han et al., 2002; Wurglics and Schubert-Zsilavecz, 2006; Perassolo et al., 2011; Huang et al., 2014). These species were known to produce substantial amount of anthraquinone derivatives. It has been reported that the mevalonic acid (MVA) and the 2-C-methyl-D-erythritol 4-phosphate (MEP) pathways produce dimethylallyldiphosphate, a precursor of the anthraquinone backbone, while the shikimate pathway produces another backbone, 1,4-dihydroxy-2-napthoyl-CoA, *via* isochorismate (Han et al., 2001; Hao et al., 2012; Peng et al., 2013). In *P. cuspidatum*, MVA, MEP, and shikimate pathways are shown to be involved in the biosynthesis of anthraquinones such as emodin and physcion (Hao et al., 2012; Peng et al., 2013). Nevertheless, the anthraquinone biosynthetic pathway in *P. cuspidatum* is largely elusive.

With the rapid development of next-generation sequencing technology, whole-genome information has been obtained for many medicinal plants. Herbgenomics is a new field of study that investigates the genetics and regulatory mechanisms of traditional Chinese herbal medicines *via* genomics, which clarifies the mechanisms of action of traditional Chinese medicines and facilitates molecular breeding from the perspective of the genome (Chen and Song, 2016; Xin et al., 2018). Herbgenomics mainly involves analysis of medicinal plant genomes, transcriptomes, proteomes, metabolomes, and so on. To date, the genomes of *Ganoderma lucidum* (Chen et al., 2012), *Salvia miltiorrhiza* (Wenping et al., 2011; Ma et al., 2012; Xu et al., 2016), *Dendrobium officinale* (Yan et al., 2015), *Andrographis paniculata* (Sun et al., 2019), *Macleaya cordata* (Liu et al., 2017), *Panax ginseng* (Xu et al., 2017), *Panax notoginseng* (Zhang et al., 2017a), *Chrysanthemum nankingense* (Song et al., 2018), and other important Chinese herbs have been described.

Dissecting the metabolic pathways of useful natural products from a genomics perspective will provide fundamental resources for the large-scale production and generation of novel chemicals *via* synthetic biology (Esvelt and Wang, 2013; Baltes and Voytas, 2015). Given that *P. cuspidatum* is a popular traditional Chinese medicine with widespread applications, the *in vivo* distributions of major components and their underlying metabolic pathways should be investigated. With the help of next-generation sequencing, genes involved in certain metabolic pathways in many medicinal plants could be identified. These approaches will not only shed light on how natural products are synthesized and how their production is regulated, but they could also be utilized for the genetic improvement of medicinal plants.

Due to their similar appearance, it is challenging to distinguish dry roots of *P. cuspidatum* from those of closely related species, such as *Fallopia multiflora* and *R. palmatum*. Traditional authentication methods such as chromatographic fingerprint analysis and spectroscopy have limited utility (Xie et al., 2006; Yap et al., 2007). DNA marker-based technology could also be used to explore intra-species genetic variation (Hon et al., 2003), which would provide important information about the relationship between genetic diversity and environmental interactions in *P. cuspidatum*. Therefore, the availability of whole-genome resources would facilitate the development of DNA marker-based technology in *P. cuspidatum*.

The lack of genomic information represents a major obstacle to exploring the biological features of *P. cuspidatum*. To address this problem, in this study, we sequenced and assembled a draft genome of *P. cuspidatum*. We annotated genes in the genome and predicted whole-genome duplication (WGD) events. Our study paves the way for the genetic analysis of *P. cuspidatum*. The draft genome produced in this study should facilitate the improvement of *P. cuspidatum*, provide a better understanding of the metabolic pathways of its natural products, give insight on its remarkable stress tolerance, and facilitate the development of methods for the biocontrol of this plant for weed management.

## Materials and Methods

### Plant Material and Sequencing

*P. cuspidatum* plants were cultivated in a field in Hubei Province, China (25°20ʹ5.496ʹʹ N, 114?57ʹ52.459ʹʹE). The fresh leaves of 1-year-old plants were collected for DNA extraction. To construct sequencing libraries, 5 μg of DNA was used. Libraries were constructed using an Illumina TruSeq DNA Preparation Kit following the manufacturer’s recommendations. The four sequencing libraries, with insert sizes of 550 bp, 2 to 3 kb, 5 to 7 kb, and 10 to 15 kb, were sequenced on the Illumina HiSeq platform (150-bp paired-end reads, PE150). In addition, 250-bp paired-end reads were generated on the Illumina HiSeq 2500 platform.

### Genome Assembly

A genome survey was performed using 67 Gb of clean Illumina sequencing data (PE150) using Jellyfish software following the instructions from the GenomeScope website (http://qb.cshl.edu/genomescope/) (Marçais and Kingsford, 2011). The genome survey indicated that this species has very high heterogeneity. Two software packages were used for genome assembly: SOAPdenovo, which is highly effective for short-read assembly, and platanus, which is thought to perform well using genomes with high heterozygosity (Luo et al., 2012). For SOAPdenovo-guided assembly, 150-bp paired-end sequencing reads were assembled into contigs, and sequencing reads with a large insertion size were used to construct scaffolds. Reads with a length of PE250 were used to fill gaps in the scaffolds with GapFiller software (Nadalin et al., 2012; Kajitani et al., 2014). For platanus-guided assembly, all sequencing reads with different insertion sizes and lengths (PE150 and PE250) were used for contig and scaffold assembly, followed by gap filling. This software was run with different Kmers, and the best assemblies from different Kmers were determined based on contig and scaffold length, gap content, and genome completeness. Due to possible sequence redundancy from the platanus assembly, Redundans software was used to filter the scaffolds (Pryszcz and Gabaldón, 2016). For scaffolds <1 kb long, BLAST analysis was performed by aligning these sequences against sequences longer than 1 kb (*E*-value < 1e-5, identity >70%, and match length >60%).

### Transposable Element Prediction

Due to the relatively low conformance of repeat sequences among species, repeat sequence databases are often constructed to predict the repeats for a specific species. By integrating four software programs, including LTR FINDER (Xu and Wang, 2007), mite-hunter (Han and Wessler, 2010), RepeatScout (Price et al., 2005), and PILER-DF (Edgar and Myers, 2005), a repeat sequence database was constructed for *P. cuspidatum* based on the principle of structural prediction and *de novo* prediction. Sequences in this database were classified using PASTEClassifier (Hoede et al., 2014) and merged with the Repbase database to form the final repeat sequence database. RepeatMasker (version 4.0.6) was then used to predict the repeat sequences in the genome based on the newly constructed database (Tarailo-Graovac and Chen, 2009).

### Gene Prediction and Annotation

Three approaches were used to predict genes in the *P. cuspidatum* genome: *de novo* prediction, homolog-based prediction, and transcriptome-based prediction. For *de novo* prediction, Augustus software (version 2.4) was utilized with a transposable element (TE)-masked genome (Stanke et al., 2006). For homolog-based approach, the GeMoMa (version 1.3.1) was used to predict genes with gene models from the other four species (three dicot species and one monocot species) (Keilwagen et al., 2016), including the dicot species Tartary buckwheat (http://www.mbkbase.org/Pinku1/), *Arabidopsis thaliana* (https://www.arabidopsis.org/) and grape (http://www.genoscope.cns.fr/externe/GenomeBrowser/Vitis/), and the monocot species rice (*Oryza sativa*) (http://rice.plantbiology.msu.edu/). For transcriptome-based prediction, the HISAT (version 2.0.4) and StringTie (version 1.2.3) programs were used for transcript assembly (Kim et al., 2015; Pertea et al., 2015). In addition, TransDecoder (version 2.0, https://github.com/TransDecoder/TransDecoder/) was used to predict gene models. All gene models obtained using these approaches were integrated with EVM software (Haas et al., 2008). To predict the putative functions of genes, all gene models were aligned against the GenBank Non-Redundant, TrEMBL, Pfam, SwissProt, Gene Ontology (GO), and Kyoto Encyclopedia of Genes and Genomes (KEGG) databases.

### Gene Family Identification and Analysis

To identify gene families based on those in other plants, OrthoMCL analysis was performed (Li et al., 2003). Protein data were downloaded from four other dicot species, including Tartary buckwheat (http://www.mbkbase.org/Pinku1/), Arabidopsis (https://www.arabidopsis.org/), grape (http://www.genoscope.cns.fr/externe/GenomeBrowser/Vitis/), and tomato (*Solanum lycopersicum*) (ftp://ftp.solgenomics.net/tomato_genome/annotation/ITAG3.2_release/). All data were subjected to BLASTp analysis (*E*-value < 1e-5), and the resulting data were grouped into gene families with OrthoMCL. To predict the expansion and contraction of the gene families and to infer lineage-specific gene gains and losses, the five genomes were analyzed using OrthoFinder v.2.2.6 (Emms and Kelly, 2015) and CAFE v.4.2.1 (De Bie et al., 2006) with default parameters. A dated species tree was downloaded from the TimeTree database (Kumar et al., 2017) and used as a guide tree. The birth-death parameter (lambda) was estimated using orthogroups in which no more than 100 genes were derived from a single genome.

### Identification of Species-Specific Genes

To explore how many genes were unique to the *P. cuspidatum* genome, gene models were downloaded from 79 plant species, and the protein data for *P. cuspidatum* were compared with data from the 79 species by BLASTp (*E*-value < 1e-5). Only genes with no homologs in the 79 other species were retained; these genes were defined as species-specific genes.

### RNA-Seq Data Analysis

The roots and mixed samples of aboveground tissues of 1-year-old plants were collected in three biological replicates, immediately frozen in liquid nitrogen, and stored at −80°C before an RNA extraction. The RNA-Seq libraries were constructed using an Illumina HiSeq Library Preparation Kit (Li et al., 2018). After validating the libraries, they were sequenced on the Illumina HiSeq platform. Trimmomatic software was used to filter out sequencing adapters and low-quality bases (Bolger et al., 2014). The clean data were mapped to the *P. cuspidatum* genome using HISAT, and gene expression levels were estimated using StringTie software (Kim et al., 2015; Pertea et al., 2015).

### Phylogenetic Analysis

To identify homologous genes in different species, BLASTp analysis was performed using Arabidopsis query sequences (*E*-value < 1e-20). The results were manually checked based on gene annotation information. Partial genes with incomplete assembly or representing pseudogenes were excluded from further analysis. The protein sequences were aligned with ClustalX software (Chenna et al., 2003) and subjected to phylogenetic analysis with MEGA X software (Kumar et al., 2018). A phylogenetic tree was constructed using the neighbor-joining method with 500 bootstrap replicates.

### Validation of RNA-Seq Data by Quantitative Real-Time RT-PCR (qRT-PCR)

qRT-PCR was performed as described previously with minor modification (Jiao et al., 2019; Zhang et al., 2019). Briefly, total RNA was isolated from roots and a mixture of aboveground tissues using Tranzol (Transgene) based on the manufacturer’s protocol (Li et al., 2019). cDNA was prepared using a PrimeScript RT Reagent Kit (RR047A, Takara). Relative expression levels were determined by qRT-PCR using the ABI 7500 Real-Time PCR system (Life Technologies). Three biological replicates and three qRT-PCR technical replicates were performed for each sample. The primer sequences used for qRT-PCR are listed in **Supplementary Table S1**.

### Extraction and HPLC Analysis of Resveratrol and Emodin

After harvest, aboveground tissues and root tissues were collected and immediately dried in an oven at 105°C for 15 min, followed by 80°C until they were completely dry. Powdered dry samples were used for HPLC analysis using a SHIMADZU LC-20AT liquid chromatograph with a model SIL-10AF autosampler injector. The HPLC was equipped with a SPD-20A programmable multiwavelength ultraviolet (UV) detector. An InertSustain C18 column (250 mm × 4.6 mm, 5 µm) with a suitable guard column (C18, 7.5 mm × 4.6 mm, 5 µm) was used. The mobile phase consisted of deionized water and acetonitrile using the following gradient program: 28% for 0–7 min, 72% for 7–9 min, and 28% for 9–20 min, with a flow rate of 1.2 ml/min and column temperature of 25°C.

## Results and Discussion

### Genome Sequencing, Assembly, and Scaffolding

**Figure 1:**
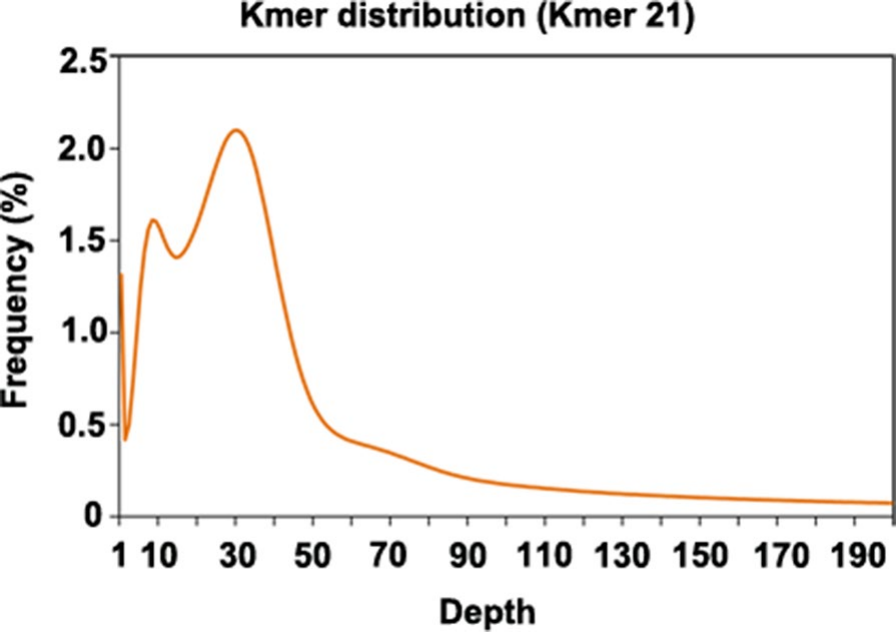
Kmer distribution of the genome survey result. The *x*-axis shows the depth of each Kmer, and the *y*-axis shows the frequency of each Kmer. In this analysis, all Kmers were 21 bp long.

Since genome information for *P. cuspidatum* was not available, we sequenced 102 Gb of Illumina data to estimate the size and heterozygosity of the *P. cuspidatum* genome *via* genome survey analysis (**Figure 1**, **Supplementary Table S2**). The *P. cuspidatum* genome size is ca. 2.6 Gb. The Kmer distribution (**Figure 1**) indicates that the *P. cuspidatum* genome contains a large proportion of repeat sequences and high heterozygosity. The genome heterozygosity was estimated to be ca. 1.6%, while the GC content was predicted to be 37.1% (**Supplementary Table S2**). These results indicate that the *P. cuspidatum* genome is highly complex, making *de novo* assembly quite challenging.

Based on the genome survey, we constructed four libraries with different insertion lengths for sequencing, including insertion sizes of 550 bp, 2–3 kb, 5–7 kb, and 10–15 kb. The libraries were sequenced on the Illumina platform (150-bp paired-end reads, PE150). We also sequenced PE250 data for genome assembly. In total, we generated 377 Gb of short-read data (**Supplementary Table S3**). To conduct *de novo* assembly of *P. cuspidatum*, we used two different software programs, SOAPdenovo (Luo et al., 2012) and platanus (Kajitani et al., 2014). The assemblies from platanus were more complete than those generated using SOAPdenovo, which is consistent with the observation that platanus performs better for highly heterozygous genomes (Kajitani et al., 2014). Following contig and scaffold assembly, we performed gap filling and removed redundant reads. The final assembled genome was 2.56 Gb long, including 948,118 scaffolds (**Table 1**). We performed BUSCO analysis (**Supplementary Table S4**) using a plant genome dataset (Simão et al., 2015), finding that our assembly covered 76.1% of *P. cuspidatum* genes. These results indicate that our assembly should be referred to as a draft genome sequence, with a large number of scaffolds and assembly gaps. Nevertheless, this study represents the first attempt to assemble the *P. cuspidatum* genome, providing a valuable resource for the community.

**Table 1:** Summary of the genome assembly and annotation in *P. cuspidatum*.

Total assembly size (bp)	2,565,149,001
Total gap length (bp)	57,172,525
Total number of scaffolds	948,118
Scaffold N50 (bp)	3,215
Max scaffold length (bp)	649,261
Total number of contigs	1,078,298
Contig N50 (bp)	2,769
Max contig length (bp)	628,071
GC content (%)	37.46
TE content (%)	71.54
Total number of genes	55,075

### TE and Functional Gene Annotations

Based on the genome assembly described above, we annotated the TEs in the *P. cuspidatum* genome. Based on this analysis, 71.54% of the sequences in the *P. cuspidatum* genome are TEs (2.0 Gb). Of these sequences, 55% were classified as class I TEs, including 30.68% *gypsy*-type long terminal repeats (LTRs), 9.88% copia LTRs, and 10.67% large retrotransposon derivatives (LARD) (**Supplementary Table S5**), whereas 4.59% of the sequences were classified as class II repeats. The proportions of these categories of TE sequences in the *P. cuspidatum* genome are in agreement with the finding that class I TEs comprise the largest proportion of all TEs in other major plant species (Quesneville et al., 2005; Flutre et al., 2011).

After masking these annotated TE sequences, we performed genome-wide gene prediction analysis of *P. cuspidatum*. To ensure accurate gene prediction, we conducted comparative transcriptome analysis between tissues sampled from roots and aboveground plant parts using a combination of *de novo* gene prediction, homology-based prediction, and transcriptome-based prediction. The results from the three approaches were integrated to constitute the final gene dataset (**Supplementary Table S6**). In total, 55,075 genes were predicted in the draft genome. Detailed characterization of the genes suggested that each gene possesses an average of four exons with an average length of 223 bp.

**Figure 2:**
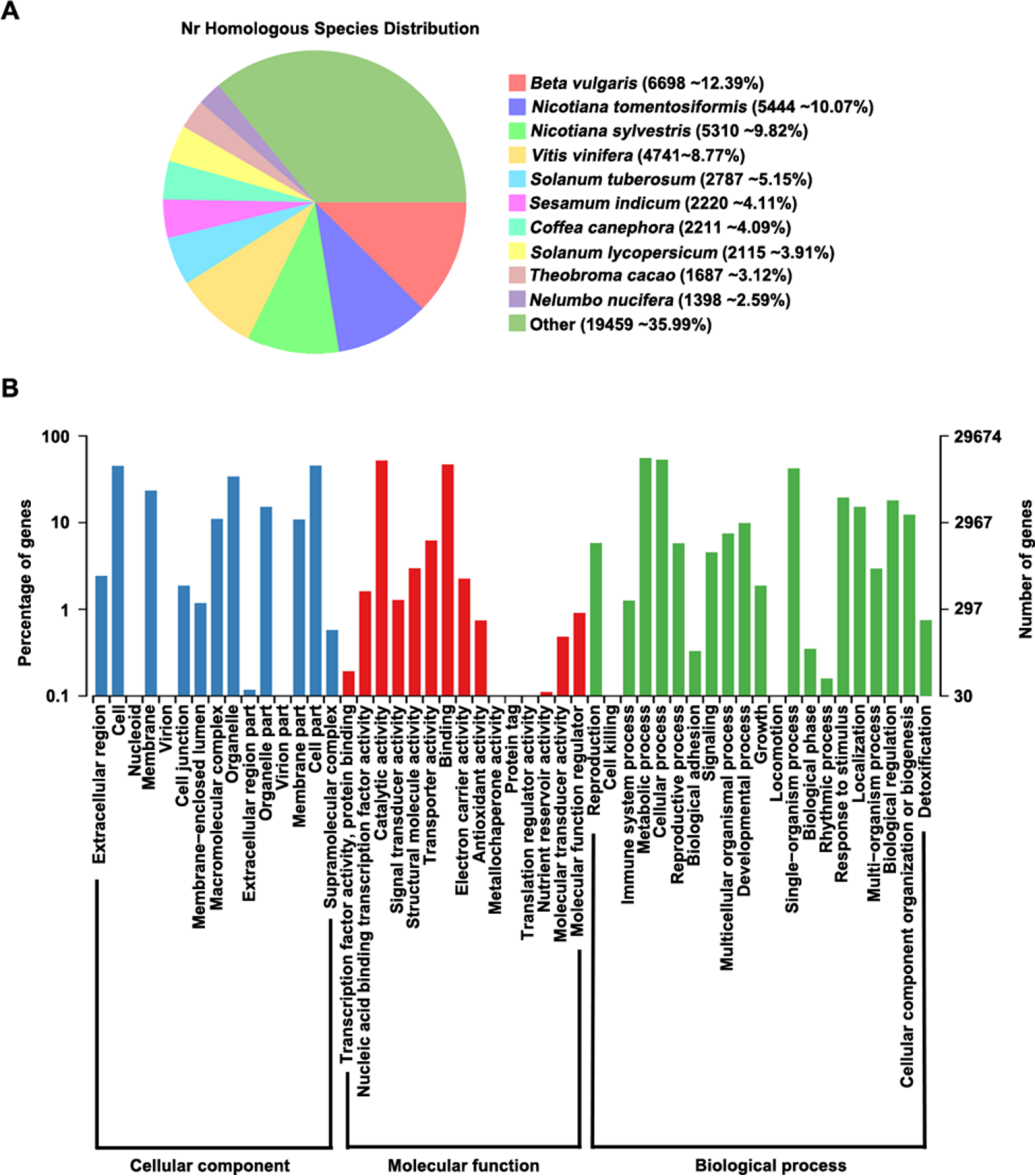
Gene annotation in *P. cuspidatum*. **(A)** Sequence alignment of all genes against the nr database. The proportions of genes with the closest homologs in different species are shown. **(B)** GO annotation of genes in *P. cuspidatum*. The GO terms were categorized into three different groups, including cellular component, molecular function, and biological process.

We aligned all predicted proteins against several databases to perform functional prediction, finding that 98.3% (54,155/55,075) of the genes could be annotated in at least one database (**Supplementary Table S7**). According to analysis of homologous genes in the GenBank Non-Redundant (nr) database, *Beta vulgaris* contains the highest proportion (12.39%) of these genes among species examined, indicating that *P. cuspidatum* and *B. vulgaris* share many homologous genes (**Figure 2A**). In addition, 29,674 genes could be assigned to at least one GO term in the categories: cellular component, molecular function, and biological process (**Figure 2B**). In the cellular component category, the most highly enriched GO terms were cell, membrane, organelle, and cell part. Two large groups of genes in the molecular function category were assigned to the GO terms “catalytic activity” and “binding,” indicating that *P. cuspidatum* contains versatile regulators of metabolite production. Consistently, most genes in the biological process category were assigned to GO terms “cellular process” and “metabolic process.”

Transcription factors (TFs) play important roles in regulating secondary metabolism, development, and environmental responses in plants. Through a comparison with *Arabidopsis*, we identified 4,753 unigenes as putative TF genes (**Supplementary Table S8**). Of the annotated TFs, 2,075 (44%) were expressed [fragments *per* kilobase of transcript *per* million mapped reads (FPKM) > 0.3] in at least one tissue, including 52 TF genes specifically expressed in roots (FPKM of roots >2 and FPKM of aboveground tissues <0.3) and 132 specifically expressed in aboveground tissues (FPKM of aboveground tissue >2 and FPKM of roots <0.3) (**Supplementary Table S8**).

### Classification of Gene Families and Characterization of Species-Specific Genes

**Figure 3:**
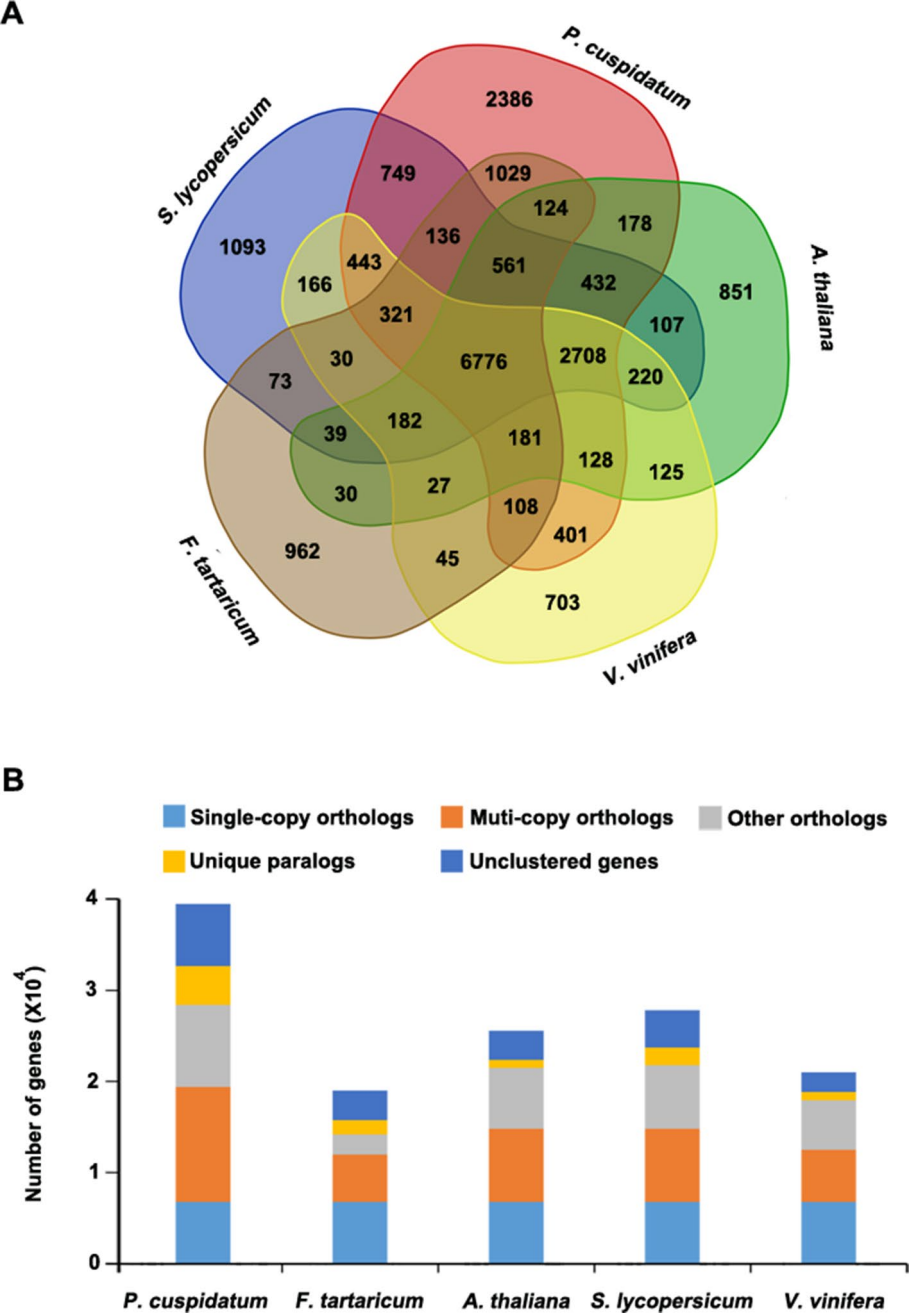
Classification of gene families. **(A)** Venn diagram showing the number of gene families in five plant species. Each number represents the number of gene families in each species or those shared by different species. The analysis was carried out using OrthoMCL software. **(B)** Summary of the number of genes in different groups. The genes were parsed from OrthoMCL clustering analysis.

**Figure 4:**
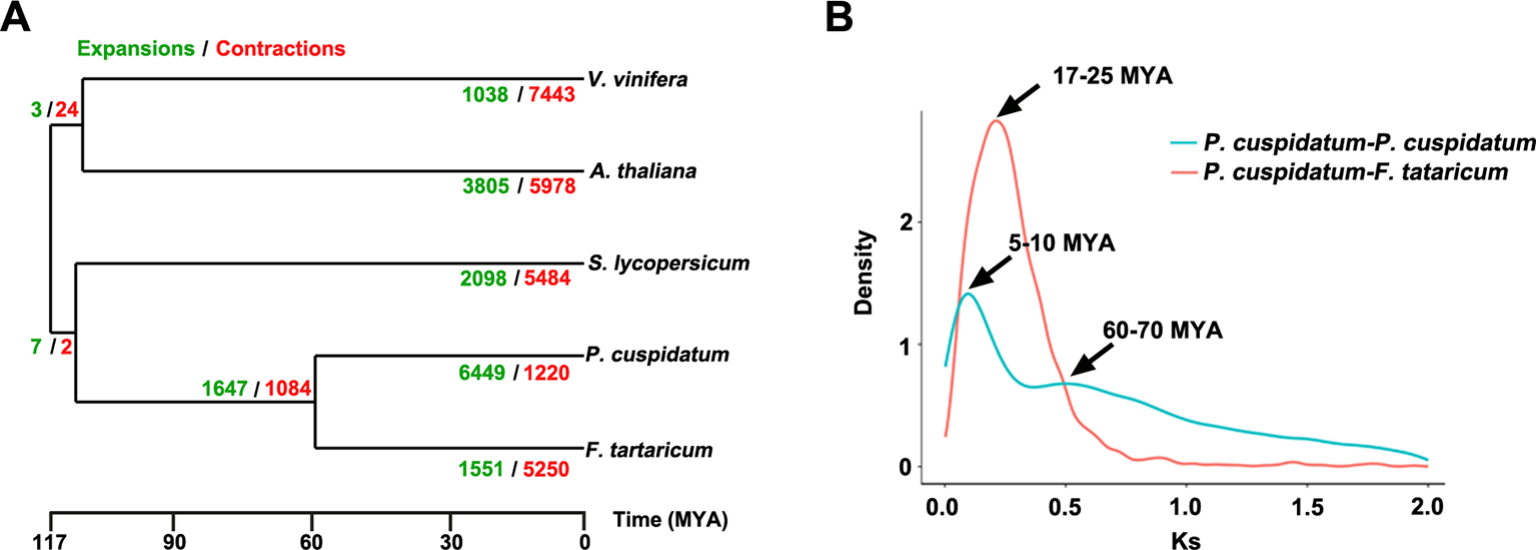
Dynamic evolution of gene families. **(A)** Gene family expansion and contraction in five plants species. The gene families that have undergone expansion and contraction are shown in green and red, respectively. The numbers separated by slashes (expansions/contractions) indicate the number of gene families. The scale bar indicates the divergence time [million years ago (MYA)]. **(B)** Ks distribution of paralogous gene pairs in *P. cuspidatum* and *F. tataricum*. In this analysis, two peaks were identified, which are thought to represent two WGD events. The *x*-axis shows Ks values, and the *y*-axis shows the density of distribution.

To uncover functional gene families in *P. cuspidatum*, we compared all genes with those of four other species, including *F. tataricum*, *S. lycopersicum*, *Arabidopsis*, and *V. vinifera*. Among these, *F. tataricum* (Tartary buckwheat) is the most closely related species to *P. cuspidatum*, which also belongs to the Polygonaceae family. The reference genome of Tartary buckwheat was recently released (Zhang et al., 2017b). *S. lycopersicum* and *Arabidopsis* are representative eudicots and are widely studied model plants. *V. vinifera*, a well-known resveratrol-producing plant (Langcake and Pryce, 1976), is an excellent plant for genome duplication analysis since it experienced no duplication events after the gamma hexaploidization (Martin et al., 2010). OrthoMCL analysis (Li et al., 2003) categorized the *P. cuspidatum* genes into 16,661 families/clusters, 6,776 of which were shared by all five species (**Figure 3A**). By contrast, 2,386 families were unique in *P. cuspidatum*, which is larger than the number of unique gene families in any of the other species. We conducted detailed analysis of genes in these families, including 39,491 genes in *P. cuspidatum*. A total of 6,776 genes in each species were clustered as single-copy orthologs. Notably, *P. cuspidatum* had the largest number of multicopy orthologs among the five species. However, 6,817 *P. cuspidatum* genes were not clustered with genes from any of the other species (**Figure 3B**). Collectively, these results indicate that *P. cuspidatum* harbors many species-specific genes. Finally, we performed expansion and contraction analysis of the gene families in these five species. Overall, the *P. cuspidatum* genome contains many contracted gene families, whereas we detected the expansion of only a few families. This pattern differs from that of other species. Among the species examined, *P. cuspidatum* had the lowest ratio of contracted versus expanded gene families, with 1,220 contracted families and 6,449 expanded families (**Figure 4A**). Compared to the four other species, even including the closely related species *F. tataricum*, *P. cuspidatum* has experienced many more gene family expansions than contractions (6,449 expansions/1,220 contractions; **Figure 4A**), indicating that increasing numbers of gene gains and retentions have been occurring in the *P. cuspidatum* genome.

To further explore the species-specific genes in *P. cuspidatum*, we downloaded 79 publically available genomes (Goodstein et al., 2012) from various species, including 5 algae species and 74 plants, including bryophytes, ferns, gymnosperms, basal angiosperms, monocots, and dicots. We separately aligned all of the *P. cuspidatum* genes to each of the 79 genomes, finding that 1,159 genes had no homologs in any of the species (**Supplementary Table S9**; *E*-value < 1e-5). By integrating our RNA-Seq data, we determined that 509 genes were upregulated in roots compared to aboveground tissues (**Supplementary Table S10**), suggesting that these species-specific genes might play distinct roles in the corresponding tissues. Of these species-specific genes, 824 (71%) were annotated as genes of unknown function. Interestingly, we identified two gene families reported to be involved in stress responses, including seven conserved abscisic acid and water-deficit stress (ABA/WDS)-induced genes (EVM0009225, EVM0028174, EVM0029800, EVM0030471, EVM0042890, EVM0043230, and EVM0051394) that play vital roles in responses to abiotic stresses (such as water-deficit, salt, and cold stress) during senescence and fruit development (Çakir et al., 2003; Liu et al., 2019; Qu et al., 2019), as well as 14 cysteine-rich transmembrane module (TM) stress tolerance genes (EVM0004810, EVM0007286, EVM0010978, EVM0012820, EVM0019558, EVM0023411, EVM0025148, EVM0025816, EVM0026927, EVM0038117, EVM0038295, EVM0042574, EVM0044873, and EVM0047701) (Bhosale et al., 2018; Xiao et al., 2018). These results are consistent with the observation that *P. cuspidatum* has high levels of abiotic stress tolerance and that many stress factors induce the accumulation of resveratrol (Hasan and Bae, 2017), providing a molecular understanding of the interaction between *P. cuspidatum* and its growth environment. Our characterization of species-specific genes provides a valuable resource for further investigating these biological processes.

### WGD Analysis

The genomes of flowering plants have undergone multiple WGD events with profound effects on genome organization, gene duplication, and fractionation (Zhang et al., 2017c). Whether the *P. cuspidatum* genome has undergone duplication events has not been previously addressed. Our assembly of the draft genome sequence of *P. cuspidatum* provides the opportunity to trace genome duplication events compared to other flowering plants. By performing all-versus-all sequence alignment, we identified 28,786 homologous gene pairs in *P. cuspidatum*, which allowed us to calculate the synonymous mutation rates (Ks) between single-copy collinear paralogous genes of *P. cuspidatum* and the closely related species *F. tataricum* at the whole-genome level. The analysis of Ks values revealed two peaks of distribution in *P. cuspidatum* (**Figure 4B**), suggesting that two WGD events have occurred approximately 6.6 million years ago (MYA) and 65 MYA. A comparison with *F. tataricum* (Zhang et al., 2017c) suggested that these plants shared a common WGD event at ca. 65 MYA. To further investigate the divergence time between *P. cuspidatum* and *F. tataricum*, we calculated the Ks values for single-copy genes *via* OrthoMCL analysis (**Figure 4B**). We identified a Ks peak at ∼21 MYA, which represents a speciation event between *P. cuspidatum* and *F. tataricum*. Collectively, these results indicate that *P. cuspidatum* had a WGD at 65 MYA, supporting the notion that many plant species shared a common WGD event at 65 MYA, which has facilitated plant survival during this period (Fawcett et al., 2009). After its divergence from a common ancestor with *F. tataricum* 21 MYA, *P. cuspidatum* experienced lineage-specific WGD at 6.6 MYA. This WGD analysis lays the foundation for genome evolution studies of *P. cuspidatum* in the future.

### Characterization of Genes That Are Preferentially Expressed in Roots

Although crude *P. cuspidatum* root tissue serves as an effective agent in traditional Chinese medicine and a major source of bioactive metabolite derivatives (for instance, resveratrol and emodin), little is known about the regulation of the biosynthetic pathways of these compounds. We therefore investigated genes that are preferentially expressed in *P. cuspidatum* roots on a genome-wide scale by conducting comparative transcriptome analysis between roots and aboveground tissues based on genome assembly and gene annotation information.

**Figure 5:**
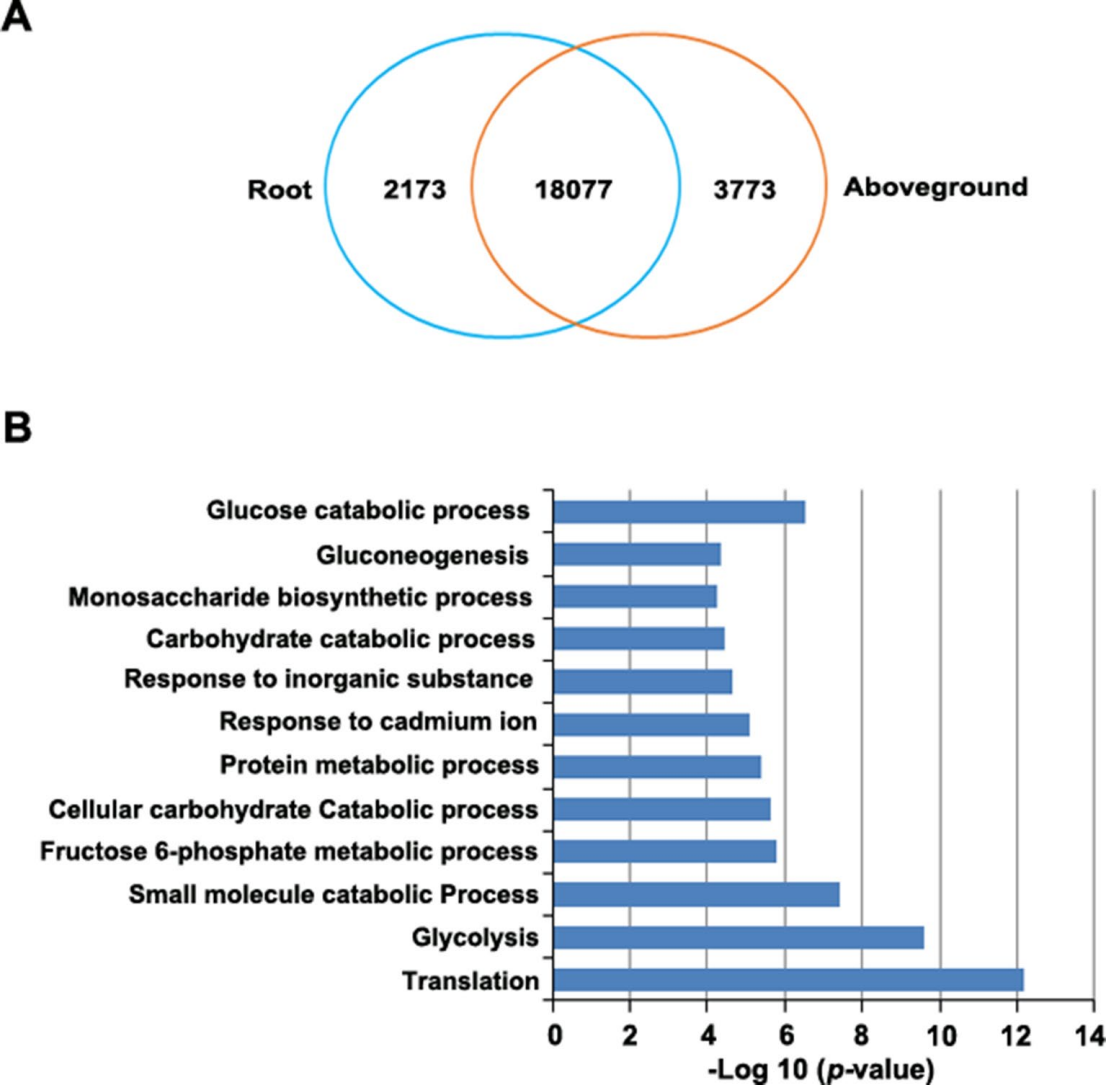
Identification of differentially expressed genes in roots versus aboveground tissues. **(A)** Venn diagram of upregulated genes in roots and aboveground tissues. The number in the overlapping region represents the number of genes that were expressed (FPKM > 0.1) in at least one sample but did not appear to be differentially expressed. The other numbers represent the number of genes that were upregulated in each sample. **(B)**, GO enrichment analysis of genes that were upregulated in root samples. The most highly enriched GO terms in different biological process categories are shown.

RNA-Seq revealed 21,415 genes that were expressed in roots, 23,100 that were expressed in aboveground tissues (FPKM > 0.1), and 20,492 that were expressed in both tissues. Of these, 923 genes were expressed only in roots and 2,608 genes were expressed only in aboveground tissues (**Supplementary Table S11**). Finally, 24,023 genes were differentially expressed between tissues, including 2,173 genes that were upregulated in roots and 3,773 that were upregulated in aboveground tissues (**Figure 5A**, **Supplementary Table S11**).

To predict the functional roles of differentially expressed genes (DEGs), we performed GO enrichment analysis. DEGs that were upregulated in roots were highly enriched in several important biological processes, such as in glycolysis and gluconeogenesis and in the monosaccharide biosynthetic process, the fructose 6-phosphate metabolic process, and glucose catabolic process (**Figure 5B**, **Supplementary Table S12**). This observation is not unexpected since various phytochemicals, such as resveratrol and anthraquinone, are important for *P. cuspidatum* roots (Zhang et al., 2013). By contrast, most upregulated genes in aboveground tissues were enriched in GO terms associated with various fundamental pathways, such as photosynthesis (**Supplementary Table S12**).

Since TFs play important roles in regulating basic biological processes, we investigated TF genes that are specifically expressed in roots to determine whether they function in the regulation of root development in *P. cuspidatum*. Several important TF families were identified (**Supplementary Table S8**), such as WRKYs (4), which play roles in abiotic and/or biotic stress responses; MYBs (5), which are important regulators of metabolite biosynthesis; lateral organ boundary domain (LBDs) (5), which are involved in lateral root development; NAM, ATAF, and CUC (NACs) (3) and APETALA2/ethylene responsive factors (AP2/ERFs) (7), which regulate the formation of ground tissues in roots; basic helix–loop–helix (bHLH) (3), most of which are involved in root hair development and auxin response factors (ARFs) (1), which modulate auxin responses during root formation. These results provide a basis for further functional analysis of genes that contribute to the formation of root architecture and the production of bioactive metabolite derivatives in *P. cuspidatum* roots.

### Genes Involved in the Resveratrol Biosynthetic Pathway

**Figure 6:**
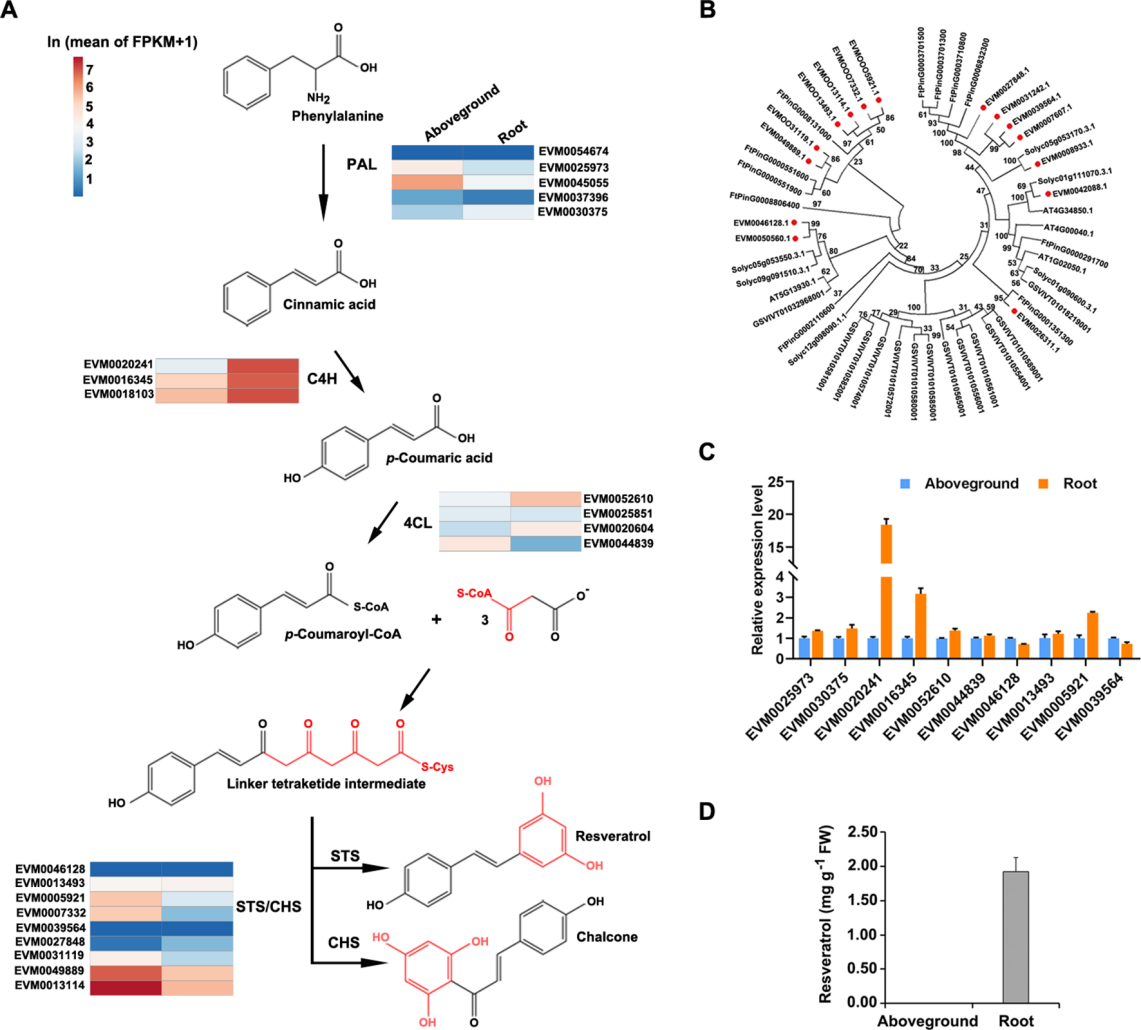
Phylogenetic and expression analysis of gene families involved in the resveratrol biosynthetic pathway in five species. **(A)**, Proposed pathway for resveratrol biosynthesis derived from the phenylpropanoid pathway in *P. cuspidatum*. PAL, phenylalanine ammonia lyase; C4H, cinnamic acid 4-hydroxylase; 4CL, 4-coumarate CoA ligase; STS, stilbene synthase; CHS, chalcone synthase. The colors represent the expression levels of each gene in ln(FPKM) in aboveground and root tissues. **(B)**, Phylogenetic tree of the *STS*/*CHS* gene family. Genes in *P. cuspidatum* are indicated by red dots. The tree was constructed using the neighbor-joining method with 500 bootstrap replicates. **(C)**, Expression analysis of the representative resveratrol biosynthetic genes, as determined by qRT-PCR. **(D)**, Resveratrol levels in roots and aboveground tissues. Each bar represents the mean value (*n* = 3). Error bars represent the SD.

Although proposed resveratrol biosynthetic pathways have been described for several plant species (Lu et al., 2016), and resveratrol is known to be produced *via* a metabolic pathway that largely overlaps with the phenylpropanoid pathway (**Figure 6A**), genetic information about the enzymes in *P. cuspidatum* on a whole-genome scale is still lacking. To address this issue, we identified and investigated the expression of the four known major players in the resveratrol biosynthetic pathway, including PAL, C4H, 4CL, and STS. PAL, C4H, and 4CL are members of the common phenylpropanoid pathway in plants, which synthesizes phenolic compounds, whereas STS, a member of the type III polyketide synthase STS/CHS superfamily, catalyzes the condensation of resveratrol in the final step of this pathway (Hao et al., 2012). We identified six PAL, four C4H, four 4CL, and nine STS/CHS genes in *P. cuspidatum* (**Figure 6A**; **Supplementary Table S13**). Since we could not identify the functions of novel genes in *P. cuspidatum* through complementation experiments, we performed phylogenetic analysis to compare *STS/CHS* genes with those from *S. lycopersicum*, *Arabidopsis*, *V. vinifera*, and the closely related species *F. tataricum* to elucidate their potential molecular characteristics (**Figure 6B**). All *STS/CHS* genes in *P. cuspidatum* had homologous genes in the four other species (**Figure 6**). Interestingly, a clade of *CHS* genes in *P. cuspidatum* appears to have been duplicated compared to those in *F. tataricum*. Of these genes, one gene was upregulated in roots, whereas the others were expressed at very high levels in both roots and other tissues (**Figure 6B**). The expansion of *STS/CHS* genes might be related to the importance of resveratrol and flavonoid metabolism in *P. cuspidatum* roots.

To experimentally validate the expression patterns of genes identified by transcriptome sequencing, we subjected the genes involved in resveratrol biosynthesis to qRT-PCR analysis. Several resveratrol biosynthesis genes were upregulated in roots compared to aboveground tissues, such as *C4H* (EVM0020241), *4CL* (EVM0052610), and *STS*/*CHS* (EVM0039564) (**Figure 6C**), which is consistent with the transcriptome data. These results suggest that resveratrol is primarily synthesized in root tissue. To confirm this notion, we measured the levels of resveratrol and the anthraquinone emodin in various tissues by HPLC and determined that resveratrol is mainly present in root tissue (**Figure 6D**; **Supplementary Figure S1D**). Even though some biosynthetic genes were also expressed in aboveground tissues, we could only detect resveratrol and emodin in the root tissue. This discrepancy between the activity of biosynthetic genes and their product might be caused by a common explanation that gene expression at transcriptional level sometimes doesn’t properly reflect the level of its products since there are also post-transcriptional and post-translational regulations. Alternatively, even if they might be produced at both root and aboveground tissues, resveratrol and emodin might accumulate predominately in the root tissue through unknown transportation mechanisms. These findings suggest that the resveratrol metabolic pathway is activated in *P. cuspidatum* roots.

### Genes Involved in the Anthraquinone Biosynthetic Pathway

Anthraquinones such as emodin and physcion are another group of major pharmaceutically active compounds in *P. cuspidatum* (Hong et al., 2016). The MVA, MEP, and shikimate pathways are involved in anthraquinone biosynthesis in plants (Wijnsma and Verpoorte, 1986; Han et al., 2001; Hao et al., 2012; Zhang et al., 2013). Although the detailed metabolic pathways are currently unknown, we identified 14, 14, and 16 unigenes that were assigned to these three metabolic pathways, respectively (**Supplementary Table S13**). We examined the expression levels of all genes in these pathways based on the transcriptome data (**Supplementary Figure S1**), which indicated that MVA pathway genes (e.g., EVM0022304) involved in anthraquinone biosynthesis are actively expressed in *P. cuspidatum* roots.

## Concluding Remarks

Due to the lack of genomic information about *P. cuspidatum*, little is known about this plant at the molecular level. The lack of genomic information has hindered the development of molecular markers to identify different varieties of *P. cuspidatum*, as well as investigations of the functions of key chemical components in *P. cuspidatum*. Here, we assembled a draft genome of *P. cuspidatum* based on Illumina short-read sequencing data. It is challenging to perform *de novo* assembly of large genomes with a high degree of heterozygosity. Since we determined that the *P. cuspidatum* genome is highly complex, with a high proportion of repeats and high heterogeneity, considerable effort will be needed to obtain a high-quality genome assembly of this plant in the future. Even though the draft genome sequence produced in this study contains many assembly gaps and perhaps assembly errors in highly complex regions, this study represents the first attempt to assemble such a sequence. The genome sequence obtained here should accelerate genomic and molecular studies of *P. cuspidatum*.

In this study, we identified 55,075 predicted genes in *P. cuspidatum* by integrating three approaches. These genes were categorized into gene families, including 1,159 that were identified as species-specific genes. By integrating our RNA-Seq data, we identified genes that are preferentially expressed in roots, which were predicted to be related to the unique biology of roots. For example, the *STS/CHS* gene family appears to have expanded and is preferentially expressed in roots, which might contribute to resveratrol biosynthesis. The results of this study provide a reference for future detailed analysis of the metabolic pathways in *P. cuspidatum* and could facilitate the utilization of *P. cuspidatum* as an important medical herb.

## Data Availability Statement

The raw sequence data reported in this paper have been deposited in the Genome Sequence Archive in BIG Data Center (Wang et al., 2017b; Members, 2019), Beijing Institute of Genomics (BIG), Chinese Academy of Sciences, under accession numbers CRA001939, CRA001941 that are publicly accessible at https://bigd.big.ac.cn/gsa.

## Author Contributions

CL, YZhang, and GW designed the study. LZ, YZhang, YZheng, YZhao, PH, XX, XH, QC, HL, CZ, ZH and XW performed the research. CL, LZ, KF, YZhang, and GW wrote the paper. All the authors analyzed the data, discussed the results, and made comments on the manuscript.

## Funding

This work was funded by the National Natural Science Foundation of China (31701294, 31801210, and 31771556), the Cultivating Project for Young Scholar at Hubei University of Medicine (2017QDJZR26, 2016QDJZR11, and 2016QDJZR14), the Natural Science Foundation of Hubei Provincial Department of Education (Q20182104), the Fundamental Research Funds for the Central Universities (GK201702016), Hubei Provincial Natural Science Foundation of China (2017CFB674), the Foundation of Health Commission of Hubei Province (ZY2019Q004), Open Research Fund of Key Laboratory of Medicinal Resources and Natural Pharmaceutical Chemistry Ministry of Education (2019005), Fund for Key Laboratory Construction of Hubei Province (2018BFC360, WLSP201905), Hubei Provincial Outstanding Young and Middle-aged Science and Technology Innovation Team Project (Grant No. T201813), the Scientific and Technological Project of Shiyan City of Hubei Province (18Y06) and the Hubei Provincial Technology Innovation Project (2017ACA176).

## Conflict of Interest

The authors declare that the research was conducted in the absence of any commercial or financial relationships that could be construed as a potential conflict of interest.
