# Supplementary material for: Assembly and Annotation of a Draft Genome of the Medicinal Plant Polygonum cuspidatum
Source: Front Plant Sci. 2019 Oct 18;10:1274. doi: 10.3389/fpls.2019.01274 (PMC6813658; doi:10.3389/fpls.2019.01274)
Supplement: Supplementary file 1 [file DataSheet_1.docx]

**Table S1 List of primers used in this study.**

| **Primer name** | **Sequence** | **Purpose** |
| --- | --- | --- |
| PAL_EVM0025973.1-qPF | CCAGAAGATTGGAGCGTTCG |  |
| PAL_EVM0025973.1-qPR | TGGATCCTTGTTGCTTCCACT |  |
| PAL_EVM0030375.1-qPF | TCTCCTGGTGAGGAGTTTGAC |  |
| PAL_EVM0030375.1-qPR | TCAGAACTCCCAAGTCCCAA |  |
| C4H_EVM0020241.1-qPF | CGGTCCCGTTTTTCACGAAC |  |
| C4H_EVM0020241.1-qPR | ATTCCCCCTCACCTCCTCC |  |
| C4H_EVM0016345.1-qPF | CAGAGCAAGCTACGAACCGA |  |
| C4H_EVM0016345.1-qPR | CATTCGGAGGCGTAGGGTTT |  |
| 4CL_EVM0052610-qPF | CCTCGTCAAGTGTCCCGATG |  |
| 4CL_EVM0052610-qPR | CCCAGAACAGCATTGGGGAG |  |
| 4CL_EVM0044839.1-qPF | CTGGCTCCACTCTCGACTTC |  |
| 4CL_EVM0044839.1-qPR | TGTGGCAGCGTCATCGTTTA |  |
| CHS_EVM0005921.1-qPF | CGCCCAAAAGGCGATCAAAG |  |
| CHS_EVM0005921.1-qPR | GGAGCTTCGTGAGCTGGTAG |  |
| CHS_EVM0039564.1-qPF | TGGGCTCAATGTCCATCTCTC |  |
| CHS_EVM0039564.1-qPR | CCAGAATAGCCTTGCCACCG |  |
| STS_EVM0046128.1-qPF | CGCACTCATCGTTGGTTCAG |  |
| STS_EVM0046128.1-qPR | CCATCAATAGCCCCACTGCT |  |
| STS_EVM0013493.1-qPF | TGTGTGCCTACATGGCTTCAT |  |
| STS_EVM0013493.1-qPR | CCTTGATGGCCTTTTGAGCC |  |
| Actin_EVM0025557.1-qPF | GAGCTTGAAACCGCCAAGAG | Reference gene |
| Actin_EVM0025557.1-qPR | CAGGGCATCTGAACCTCTCA |  |

**Table S2 Raw data from genome survey analysis.**

| Sample | Raw clusters | Raw yield (Mbases) | GC (%) | Q30 (%) |
| --- | --- | --- | --- | --- |
| J Knotweed | 339370896 | 102,490 | 37.10 | 89.65 |

**Table S3 Summary of sequencing data.**

| library | # of read pairs | data size (bp) | data size (Gb) |
| --- | --- | --- | --- |
| 550 bp | 223,984,706 | 67,195,411,800 | 67 |
| 2-3 Kb | 501,312,857 | 150,393,857,100 | 150 |
| 5- 7 Kb | 254,146,797 | 76,244,039,100 | 76 |
| 10-15 Kb | 280,062,263 | 84,018,678,900 | 84 |
| Total | 1,259,506,623 | 377,851,986,900 | 377 |

**Table S4 Summary of BUSCO analysis using the platanus program.**

| Database | Number of gens | Percentages (%) |
| --- | --- | --- |
| Complete BUSCOs (C) | 1096 | 76.1 |
| Complete and single-copy BUSCOs (S) | 858 | 59.6 |
| Complete and duplicated BUSCOs (D) | 238 | 16.5 |
| Fragmented BUSCOs (F) | 149 | 10.3 |
| Missing BUSCOs (M) | 195 | 13.6 |
| Total BUSCO groups searched | 1440 | 100.0 |

**Table S5 Summary of transposable element annotation.**

| **Type** | **Number** | **Length（bp）** | **Percentage (%)** |
| --- | --- | --- | --- |
| ClassI | 2,986,574 | 1,540,303,859 | 55.06 |
| ClassI/DIRS | 101,877 | 69,520,500 | 2.49 |
| ClassI/LINE | 206,399 | 84,208,968 | 3.01 |
| ClassI/LTR/? | 25,375 | 14,055,292 | 0.5 |
| ClassI/LTR/Copia | 491,066 | 276,483,963 | 9.88 |
| ClassI/LTR/Gypsy | 1,201,451 | 858,377,090 | 30.68 |
| ClassI/LTR/DIRS | 194 | 21,399 | 0 |
| ClassI/PLE/LARD | 757,220 | 298,585,358 | 10.67 |
| ClassI/SINE | 29,566 | 5,272,989 | 0.19 |
| ClassI/SINE/TRIM | 1,305 | 250,738 | 0.01 |
| ClassI/TRIM | 149,153 | 30,875,920 | 1.1 |
| ClassI/Unknown | 22,968 | 6,393,358 | 0.23 |
| ClassII | 324,854 | 128,398,709 | 4.59 |
| ClassII/Crypton | 50 | 2,826 | 0 |
| ClassII/Helitron | 12,591 | 4,305,193 | 0.15 |
| ClassII/MITE | 75,686 | 16,051,222 | 0.57 |
| ClassII/Maverick | 3,390 | 1,673,485 | 0.06 |
| ClassII/TIR | 202,993 | 99,000,005 | 3.54 |
| ClassII/Unknown | 30,144 | 9,894,680 | 0.35 |
| Potential Host Gene | 8,915 | 2,476,032 | 0.09 |
| SSR | 48,603 | 9,992,492 | 0.36 |
| Unknown | 2,050,441 | 474,277,991 | 16.95 |
| Total | 3,368,946 | 2,001,537,828 | 71.54 |

**Table S6 Summary of gene predictions by different approaches.**

| **Method** | **Software** | **Species** | **Gene number** |
| --- | --- | --- | --- |
| *Ab initio* | Augustus | - | 203,268 |
| Homology-based | GeMoMa | *Arabidopsis thaliana* | 48,178 |
|  |  | *Oryza sativa* | 46,078 |
|  |  | *Vitis vinifera* | 51,060 |
|  |  | *Ipomoea nil* | 54,768 |
| RNA-seq based | TransDecoder | *-* | 97,774 |
| Integration | EVM | - | 55,075 |

**Table S7 Summary of gene predictions.**

| **#Anno_Database** | **Annotated_Number** | **100<=Protein length<300** | **Protein length>=300** |
| --- | --- | --- | --- |
| GO_Annotation | 29,674 | 17,236 | 10,562 |
| KEGG_Annotation | 21,457 | 12,464 | 7,708 |
| Pfam_Annotation | 41,831 | 22,169 | 18,034 |
| Swissprot_Annotation | 38,329 | 20,822 | 15,708 |
| TrEMBL_Annotation | 54,020 | 30,562 | 20,303 |
| nr_Annotation | 54,083 | 30,616 | 20,307 |
| All_Annotated | 54,155 | 30,662 | 20,316 |
