# Supplementary figures and images for: Assembly and Annotation of a Draft Genome of the Medicinal Plant Polygonum cuspidatum
Source: Front Plant Sci. 2019 Oct 18;10:1274. doi: 10.3389/fpls.2019.01274 (PMC6813658; doi:10.3389/fpls.2019.01274)

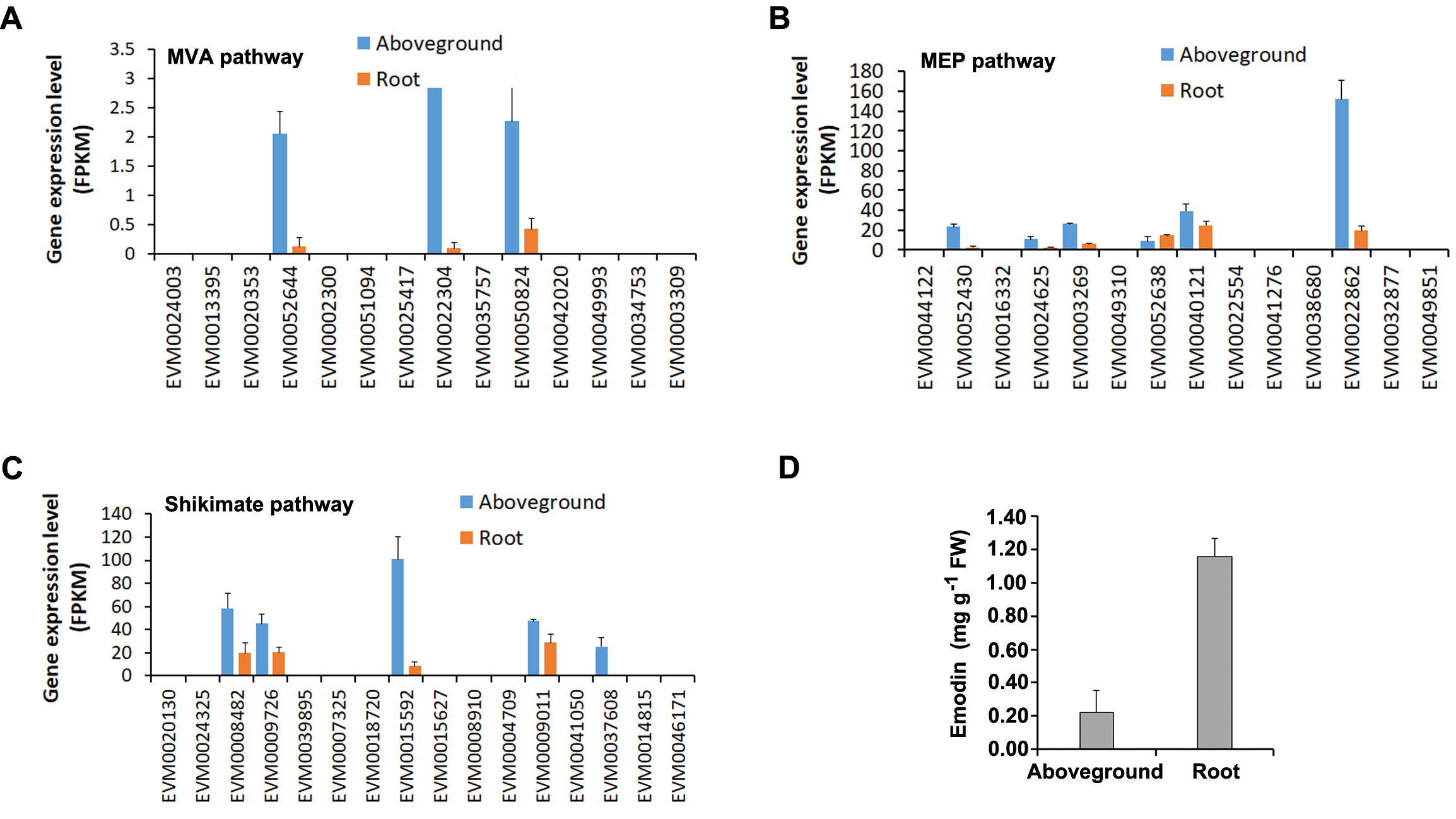

Supplement: Supplementary file 8 [file Image_1.tif]
